# Impact of Nanoparticle Uptake on the Biophysical Properties of Cell for Biomedical Engineering Applications

**DOI:** 10.1038/s41598-019-42225-7

**Published:** 2019-04-10

**Authors:** Md Alim Iftekhar Rasel, Sanjleena Singh, Trung Dung Nguyen, Isaac O. Afara, Yuantong Gu

**Affiliations:** 1 School of Chemistry, Physics and Mechanical Engineering, Queensland University of Technology (QUT), Brisbane, Australia; 2 Department of Aerospace and Mechanical Engineering, College of Engineering, University of Notre Dame, Notre Dame, Indiana 46556 USA; 3 Department of Applied Physics, University of Eastern Finland, Kuopio, Finland

## Abstract

Nanomaterials are currently the state-of-the-art in the development of advanced biomedical devices and applications where classical approaches have failed. To date, majority of the literature on nanomaterial interaction with cells have largely focused on the biological responses of cells obtained via assays, with little interest on their biophysical responses. However, recent studies have shown that the biophysical responses of cells, such as stiffness and adhesive properties, play a significant role in their physiological function. In this paper, we investigate cell biophysical responses after uptake of nanoparticles. Atomic force microscopy was used to study changes in cell stiffness and adhesion upon boron nitride (BN) and hydroxyapatite (HAP) nanoparticle uptake. Results show increase in cell stiffness with varying nanoparticle (BN and HAP) concentration, while a decrease in cell adhesion trigger by uptake of HAP. In addition, changes in the biochemical response of the cell membrane were observed via Raman spectroscopy of nanoparticle treated cells. These findings have significant implications in biomedical applications of nanoparticles, e.g. in drug delivery, advanced prosthesis and surgical implants.

## Introduction

Over the years, multiple studies have been conducted to evaluate the toxicity and interaction of nanoparticles with biological materials^1–4^. However, a substantial amount of these studies have been largely restricted to the biological consequences of nanoparticles uptake mainly based on biological assays. With the rapid increase in nanomaterial applications in various fields, it is imperative to investigate the interaction of nanoparticles with cells, including their biological as well as biophysical implications, in order to understand the extent of nanoparticle toxicity. Nanoparticles such as boron nitride (BN) and hydroxyapatite (HAP) have gained considerable interest in biomedical applications due to their properties and biocompatibility. BN possesses good lubricating properties, resistance to chemical attack and oxidation, high thermal conductivity and low thermal expansion, excellent temperature resistance and electrical insulation^5–15^. Number of studies have been conducted on the interaction of BN nanotubes with a variety of biological bodies (*e*.*g*., human neuroblastoma, mouse myoblast and human embryonic kidney)^16,17^. In comparison, studies to investigate BN nanoparticle interaction are fairly limited. On the other hand, HAP is a naturally occurring mineral abundant in human bones and teeth, and has been widely used in tissue engineering applications, such as prosthetic implants and bone replacement^18–20^. Because of their attractive properties and potential in biomedical applications, multiple studies has been conducted to evaluate the biocompatibility of both BN and HAP. In our previous study, we have studied the toxicity of BN by performing several biological assays^21^. As for HAP, multiple studies have been conducted exploring their biocompatibility^4,22^. Overall, both BN and HAP were found to be biocompatible within reasonable constrains (concentration, size, shape and morphology etc).

In this study, we hypothesize that in addition to biological responses, nanoparticle uptake may significantly affect the biophysical properties of cells. As a physical entity, cells need to maintain a degree of structural stability for normal physiological functions. For example, a healthy eukaryotic cell is expected to spread when suspended, generate adhesion to substrate over time, resist deformation under external loading, and interact with surrounding cells and extra-cellular matrix. In essence, cells possess internal mechanisms that are naturally coded to perform these functions optimally. More so, these functions are highly dependent upon the morphology and biophysical responses of the cells in their physiological environments, *e*.*g*., osteocytes possess round morphology that allows them to act as mechano-sensors in the bone, detecting and responding to different levels of force^23^. In addition, chondrocytes (cells embedded with articular cartilage matrix) possess shapes and orientations that are highly dependent upon their location within cartilage matrix, and changes in the biophysical/mechanical properties of these cells is suggested to be associated with the development and progression of osteoarthritis, a degenerative joint condition^24^. Thus, changes in the biophysical properties and responses of cells are precursors to potential alteration in tissue function. Therefore, it is crucial that the structure and biophysical properties of cells are retained under all circumstances.

In our previous study, we have demonstrated that the global mechanical property of cells remains unaffected after BN uptake^21^. However, the local mechanical property still remains unexplored. On the other hand, few studies have reported variation in adhesion strength of nanoparticle treated cells post trypsinisation^25^. These reports further emphasize the importance of exploring biophysical property changes in nanoparticle treated cells. This study aims to investigate the effect of nanoparticle uptake on the biophysical properties of cells. The mechanical (stiffness) and adhesion properties (a cell’s ability to adhere to its substrate) of human osteoblasts were quantified and compared before and after nanoparticle treatment. These properties (stiffness and adhesion) are known to play crucial roles in a number of cellular functions such as cell deformation, motility, signal transduction, migration and proliferation and therefore must be retained after nanoparticle uptake to ensure normal cell function^26,27^. Human osteoblast (OB) cells were chosen as the biological specimen of choice because they are excellent proxies and good representative of other cells in humans^28^. An improvised atomic force microscopy (AFM) setup was developed and utilised to accurately quantify the cell responses. In addition, Raman spectroscopy was adopted to identify changes in cell membrane biochemical composition, such as amides and nucleic acids; after nanoparticle uptake. It is important to note that, the effect of material property (size, shape and surface) has not been considered in this study. The outcomes of this study will shed more light on the interaction of nanoparticles with cells in terms of toxicity, and the corresponding effects on the biophysical properties and response of cells. This insight could potentially improve and optimize the use of nanoparticles and nanomaterials in biomedical applications, such as in drug delivery, advanced prosthesis and surgical implants.

## Results and Discussion

### BN and HAP nanoparticle uptake by human osteoblasts

**Figure 1:**
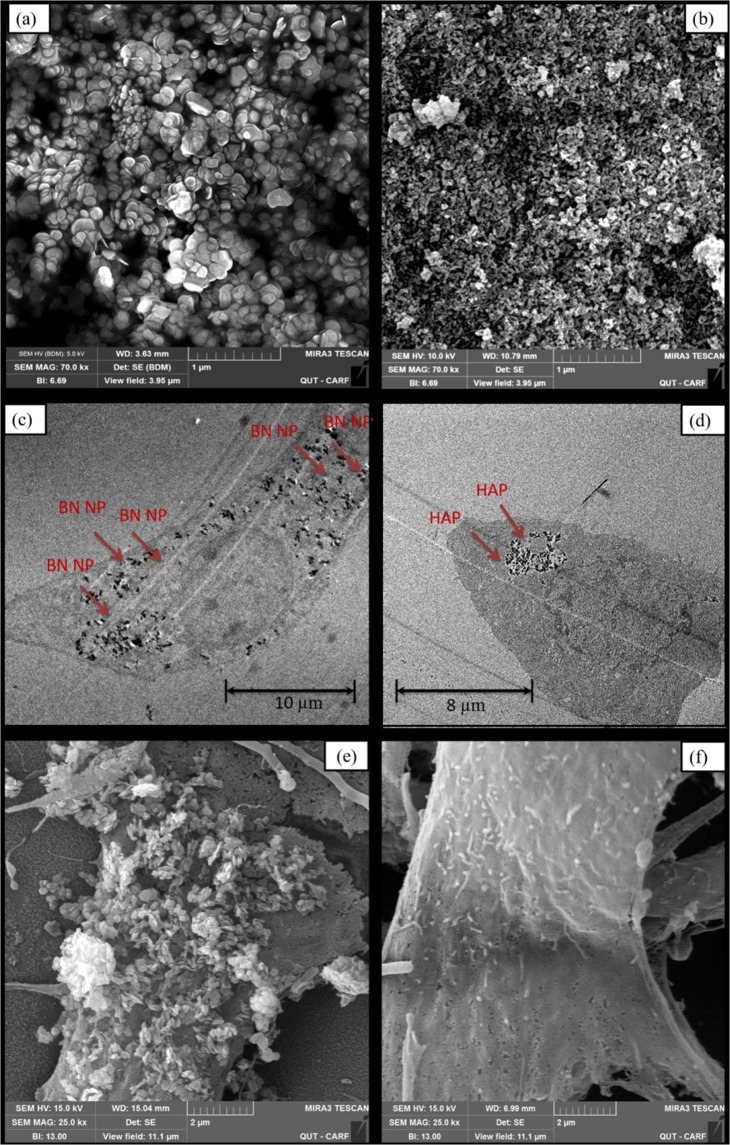
Microscopic representation of BN NP and HAP, and their uptake by human osteoblast cells, (**a**) SEM image of BN NP, (**b**) HAP. (**c**) TEM image of human osteoblast cells after culturing with BN NP, (**d**) human osteoblast cells after culturing with HAP. SEM images of cell membranes after 24 hr culture with (**e**) BN and (**f**) HAP.

Scanning electron microscopy (SEM) images reveal the approximate size and shapes of BN and HAP nanoparticles (Fig. 1a,b). BN nanoparticles were observed as single large particles with slight spherical shapes (Fig. 1a), and are between 150 and 250 nm in size. In comparison, HAPs were smaller (40–50 nm) and needle-like in shape, with a tendency to form aggregates (Fig. 1b) (also Supplementary Fig. 1). The effectiveness of nanomaterials for biomedical applications depends on successful ingestion/uptake by cells, and this was verified via transmission electron microscopy (TEM) images. Essentially, the cells were cultured in BN and HAP for 24 hrs, sectioned into thin slices (80 nm) and then imaged with TEM. The sectioning exposed the internal structures of the cell and provided evidence on the position/concentration of nanoparticles.

The TEM images show ingested BN and HAP nanoparticles within the cell cytoplasm (Fig. 1c,d), indicating successful uptake. The uptake of BN within cells observed in this study follow a similar pattern as previously reported in our earlier study^21^, whereby BN were distributed throughout the cytoplasm and the cells retained their spread-out morphology (Fig. 1c). On the contrary, a localized aggregation of HAP nanoparticles was observed, and the cells did not have a normal fibroblastic morphology (Fig. 1d). Similar effect of HAP has been observed on human hepatoma cell lines^17^ and HK-2 cells^29^ where cell proliferation was inhibited by cell membrane shrinkage, thus inducing apoptosis. An interesting observation was that nanoparticles tend to deposit around the cell cytoplasmic area, with little or no deposit in the nucleus.

SEM reveals aggregation of BN nanoparticles on the surface of cells after 24 hrs incubation (Fig. 1e). This makes it difficult to accurately quantify the amount of ingested nanoparticles. This has significant implications in applications where an accurate determination of nanoparticle dosage is critical, such as in dose-dependent drug delivery. In contrast, cells incubated in HAP exhibited smoother cell membrane without any nanoparticle deposition (Fig. 1f), possibly due to the smaller and less aggregated particles on the surface. However, HAP nanoparticles have been shown to exhibit toxicological effect on pulmonary surfactant lining by depleting surface proteins and the phospholipids on membrane layer^30^.

### Effect of nanoparticle uptake on cell biophysical properties

**Figure 2:**
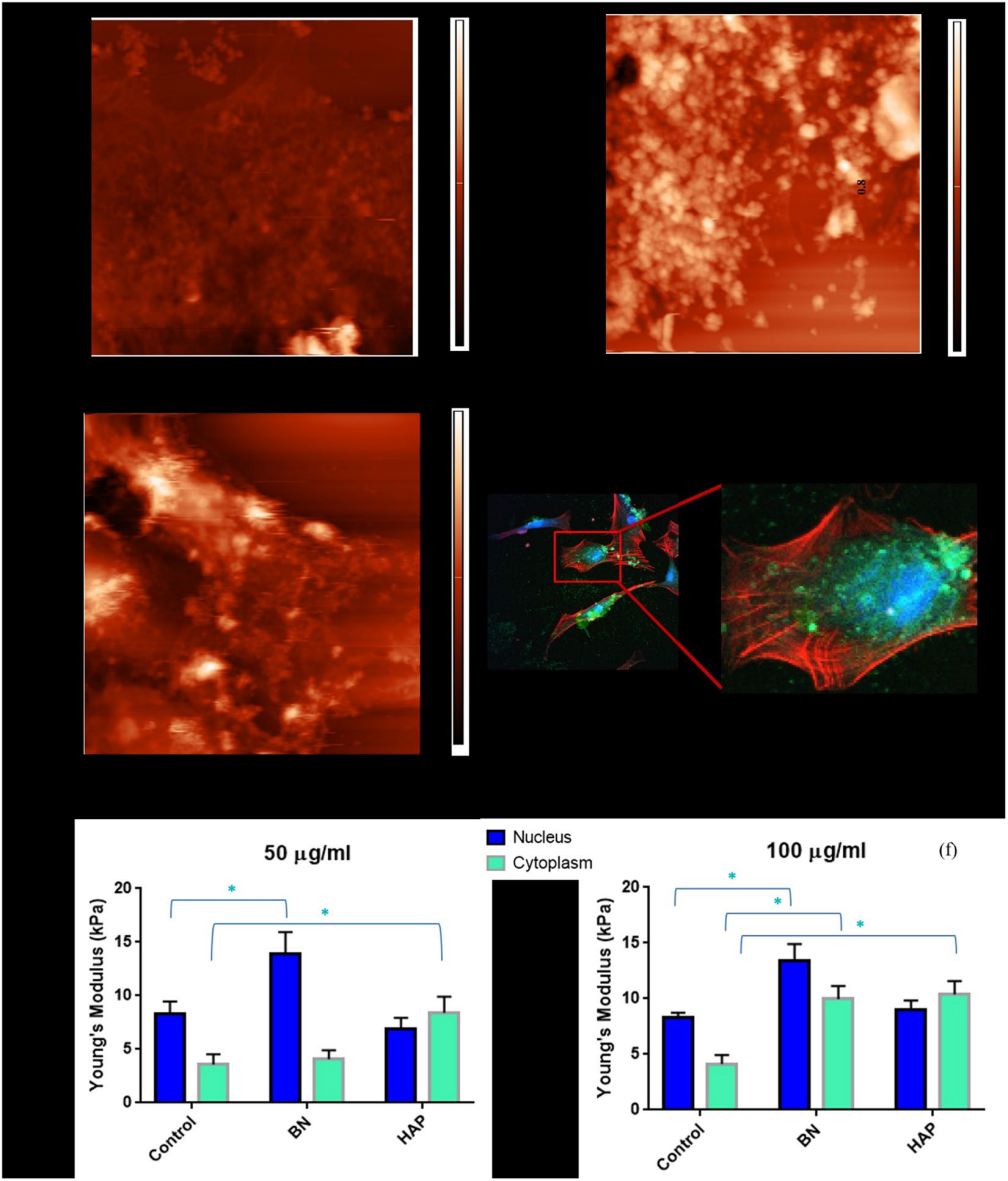
Mechanical properties of human osteoblast cells obtained through nano-indentation using AFM. AFM image of (**a**) normal (**b**) 24 hrs BN nanoparticle cultured and (**c**) 24 hrs HAP nanoparticle cultured human osteoblast cells. The Young’s modulus of cells cultured in BN and HAP (**d**) at 50 µg/ml concentration and (**e**) 100 µg/ml concentration (p < 0.05). (**f**) Confocal image of human osteoblast cells cultured in BN nanoparticle for 24 hrs at 25 µg/ml concentration. BN are indicated as green, and the F-actin is stained red (wit alexa fluor 568).

The biophysical properties of cells are mainly dictated by the cell membrane, which retains the cell shape, provides necessary support and protects intracellular components. External perturbations from materials like BN and HAP often alter the normal functions of living cells and trigger a variety of time dependent biochemical changes. Usually, these changes originate from membrane receptor signalling, which eventually leads to nuclear fragmentation and cytoplasmic shrinkage^31^. To assess the cell membrane response, as well as to gain further insight into the interaction between the nanoparticles and cell surface, AFM was utilised to probe the cell membrane response after nanoparticle uptake. BN and HAP treated cells exhibited less consistent membrane profiles after nanoparticle uptake (Fig. 2b,c) in comparison with normal cells (Fig. 2a), where the normal cells appear to be flat and well spread. For BN treated cells, this lack of consistency is possibly due to the BN nanoparticles aggregation on the cell surface (Fig. 1e). For HAP treated cells, the lack of consistency is likely due to disruptive biophysical changes in the cell membrane structure. In both cases, the cell membrane irregularity could also be due to concomitant biochemical changes observed via Raman spectroscopy. Though less HAPs were observed on the membrane surface, the overall profile appears somewhat distorted, (Fig. 2c) indicating possible disruptions in membrane structures.

The biophysical properties investigated in this study (mechanical property via stiffness, and adhesion property) are crucial for normal cellular functions and any unwanted alteration may result in failure. To assess the mechanical property, nano-indentation was performed and the Young’s modulus was calculated from the force-displacement curve (*F-δ*). The indentation site was carefully selected avoiding any sedimentated nanoparticle on the cell surface through live imaging. The force-displacement curve was then fitted with an accurate contact model to obtain the mechanical parameters of the sample. A number of cantilever shapes (spherical, rectangular, pyramidal etc.) can be chosen depending on the nature of the indentation and material characteristics. Traditionally, pyramidal cantilevers tips have been widely used for imaging soft materials and living cells. Recently, Rico *et al*. developed a robust model to quantify the stiffness of living cells using pyramidal cantilever tips and compared the results with spherical tips^32^. This model was found to be more robust and accurate, and was therefore adopted in this study.

Three batches of cells (20–30 cells per batch) were cultured with BN NP and HAP for 24 hrs at concentrations of 50 and 100 µg/ml. Their mechanical properties were then determined via nano-indentation using AFM. The indentation was performed in the central region (nucleus) and in the cytoplasmic area surrounding the nucleus. The indentation areas were illustrated via schematic diagram in Supplementary Materials (Fig. 2). From prior TEM images, most nanoparticles were observed to be distributed in cytoplasmic area (Fig. 1) therefore, indentation was performed to investigate their effect on cell biophysical properties. As the nucleus is surrounded by the cytoplasm, indentation was also performed on the nucleus to study any associated effects. The stiffness quantified in these tests represents local mechanical property of cell. Based on the area of indentation, stiffness is expected to vary (as the cellular components vary depending on site). In other words, the area of indentation dictates the calculated mechanical property.

In the nucleus region, the average Young’s modulus for normal (control), BN and HAP treated cells were 8.33, 13.89 and 6.93 kPa, respectively at a concentration of 50 µg/ml (Fig. 2e). No significant difference (p = 0.319) was observed in the Young’s modulus of normal and HAP treated cells. However, a significant increase (p = 0.028) was observed in BN treated cells, as BN nanoparticle uptake appears to increase the overall stiffness of the cell. In the cytoplasmic region, the Young’s modulus for normal (control), BN and HAP treated cells were recorded as 3.6, 4.08 and 8.4 kPa, respectively. An overall increase in the stiffness of HAP treated cells was observed (p = 0.014). Consistent with patterns in the nucleus region of cells treated at 50 µg/ml, the average Young’s modulus for control (normal), BN and HAP treated cells in the nucleus region were 8.33, 13.89 and 6.93 kPa, respectively at a concentration of 100 µg/ml (Fig. 2f). A significant increase in stiffness (p = 0.001) was observed in BN treated cells. In the cytoplasmic region, increase in Young’s modulus for BN (p = 0.004) and HAP (p = 0.001) treated cells were observed relative to normal cells. In contrast to 50 µg/ml treated cells, an overall increase in cellular stiffness was observed for both BN and HAP treated cells relative to normal cells. This is probably due to localisation of both nanoparticles within the cytoplasm, with little presence in the nucleus.

As hypothesised, the uptake of nanoparticles was observed to alter the local mechanical property of the cells. The cell mechanical properties are highly fine-tuned, any alteration may result in fatal consequences. The results obtained in this study also demonstrate that the mechanical properties are site-dependent post nanoparticle uptake, as inconsistencies in stiffness was observed in different regions. More so, variation in the distribution of BN and HAP nanoparticles within the cell cytoplasm may also play a role in the alteration of the overall stiffness of the cell.

A key components that dictate the stiffness of human cells are the cytoskeleton (CSK) and the intracellular fluid^33,34^. Studies have shown that once subjected to external loading, the CSK significantly alters its structure, with corresponding flow of the intracellular fluid through the matrix during deformation^35,36^. The bulk stiffness of the cell increases with ingestion of solid nanoparticles. This is followed by a corresponding disruption and rearrangement of the CSK and the intracellular fluid when subjected to external load. Essentially, the nanoparticles ‘clog’ up the CSK, altering the normal flow of intracellular fluid. This explains the increased stiffness in the cytoplasmic region for both BN and HAP treated cells.

A similar phenomenon was observed in a previous study conducted by Trung *et al*.^37^ where the increase in stiffness of fixed cells was attributed to the CSK and intracellular fluid trapping. However, the stiffness in nucleus region was also observed to increase in BN treated cells. In order to explore the underlying mechanism, the uptake of BN and their distribution in the cell surface were investigated. HAP nanoparticles form close aggregates after uptake and were generally found distributed in one or two spots in the cytoplasmic region (Fig. 1d). BN nanoparticles, on the other hand, were distributed throughout the cell cytoplasm (Fig. 1c). The distribution of BN can be further observed in the confocal image (Fig. 2d). Similar to the TEM image BN are observed to be distributed throughout the cytoplasmic region surrounding the nucleus from all direction (Fig. 2d). The distribution of BN nanoparticles makes the rearrangement of CSK and intracellular fluid distribution relatively difficult once subjected to external loading, even though no nanoparticle physically occupies the indented space. Hence, this is the likely reason for the significant increase in stiffness observed in the nucleus region of BN treated cells.

Like mechanical property, a number of fundamental biological phenomena, like mitosis, cell orientation, morphogenesis, embryogenesis etc., are highly dependent on a cell’s ability to adhere to the extracellular matrix and adjacent cells^38–42^. To assess this property, AFM was used to detach cells from their substrate in order to quantify the strength of adhesion. The lateral forces of nanoparticle treated cells were then compared with that of control cells.

In earlier studies, researchers adopted a continuous scanning approach, where the AFM tip was used to repeatedly scan the cell surface until detachment^43,44^. The major drawback of such technique is that the repeated contact between the cantilever tip and cell surface may significantly alter the cell’s adhesion property. Therefore, in this study the detachments of cells were performed in a single motion. Once the scanline of the cantilever was established, it was quickly moved to the centre of the cell (just in front of the nucleus) and detached with a single scan line (Fig. 2b). This improvisation was expected to provide a reliable estimate of the lateral force necessary to detach cells (adhesion property).

**Figure 3:**
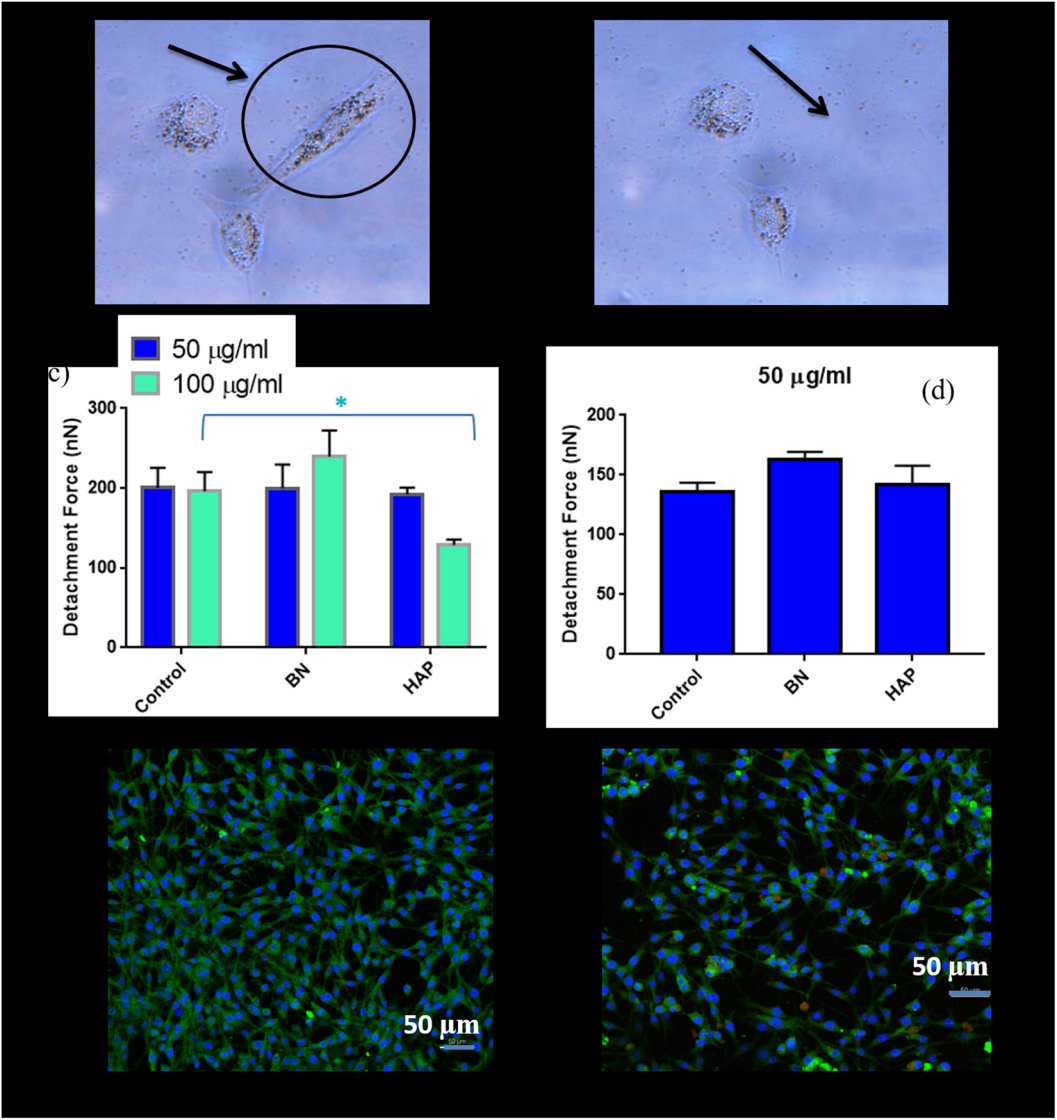
Adhesion property of human osteoblast cells obtained using AFM cantilever. (**a,b**) Microscopic image of cells before and after detachment. (**c**) Calculated detachment force of normal (control), BN and HAP treated osteoblast cells for material concentration of 50 µg/ml and 100 µg/ml. (**d**) Calculated detachment force of normal (control), BN and HAP treated osteoblast cells for material concentration of 50 µg/ml. (One way ANOVA, p < 0.05). (**e**) The focal adhesion area of normal (control) cells. (**f**) The focal adhesion area of HAP treated cell. Cells were stained with anti-vinculin determine the focal adhesion area.

The representative images of positive lateral detachment of normal, BN and HAP treated cells (cultured for 24 hrs) at a concentration of 50 µg/ml and 100 µg/ml are presented in Fig. 3c,d. At a concentration of 50 µg/ml, the average lateral forces were 200.37, 199.80 and 195.52 nN, respectively. Thus, no significant difference in adhesion strength was quantified via the lateral detachment tests (Fig. 3c),. The attachment process for all cases appears to be natural and spontaneous. At 100 µg/ml, no significant difference (196.09 and 240 nN respectively, p = 0.278) in detachment force was observed between normal and BN treated cells.

However, a significant decrease in adhesion force relative to normal cells (from 196.09 nN to 128.60 nN, p = 0.032) was observed in HAP treated cells. Thus, the uptake of HAP significantly results in a reduction in the adhesion property of cells. This phenomenon has been observed in earlier studies where significant loss of cellular adhesion after nanoparticle uptake was reported^25,45^.

In a recent study, it was observed that cells which function normally may lose their adhesion properties when trypsinised and subcultured^25^. To investigate this phenomenon on osteoblasts, the cells were treated with BN and HAP nanoparticles, trypsinised, and seeded on the petri dish for adhesion test. Since the cells adapted well at 50 µg/ml, they were cultured at this concentration. The lateral force for trypsinised normal, BN and HAP treated human osteoblasts (cultured for 24 hrs) was quantified as 136.54, 163.38 and 141.87 nN, respectively (Fig. 3d). No significant difference in lateral force was observed in all cases. Thus, nanoparticle uptake does not seem to have any significant effect on cell adhesion, even after the trypsinisation. In general, HAP nanoparticles uptake seems to affect the adhesion property of cells, particularly at higher concentration (100 µg/ml). As with the mechanical property, significant alteration in adhesive property may have dire consequences on normal cell function and should be taken into consideration in the design and applications of nanotechnology in the biomedical field.

To explore the mechanism behind the loss of adhesion, we hypothesised that HAP uptake decreases the focal adhesion area of the cell, thus decreasing the bulk cell adhesive property. To test this hypothesis, the focal adhesion area was determined by staining normal (Fig. 3e) and HAP treated cells (Fig. 3f) with anti-vinculin. Visually, no significant difference can be observed in both cases, and the cells seem to spread naturally without demonstrating any abnormal characteristics. The focal adhesion area was quantified via image analysis in imageJ (Wayne Rasband, National Institute of Health, USA). Initially, the image was converted to 8-bit grayscale, and then to a binary model. The binary model was then analysed and the adhesion area was calculated. The results are summarised in Table 1 below:

**Table-1:** The average focal adhesion area of cells.

Cell type	Count	Total area (µm^2^)	Average area (µm^2^)
Normal	543	152919	281.62
HAP 100	634	131621	207.60

No statistically significant difference was observed in the average adhesion area between normal and HAP treated cells. Thus, it can be asserted that the uptake of HAP has minimal effect on the focal adhesion area, and therefore the bulk adhesive property of cells.

**Figure 4:**
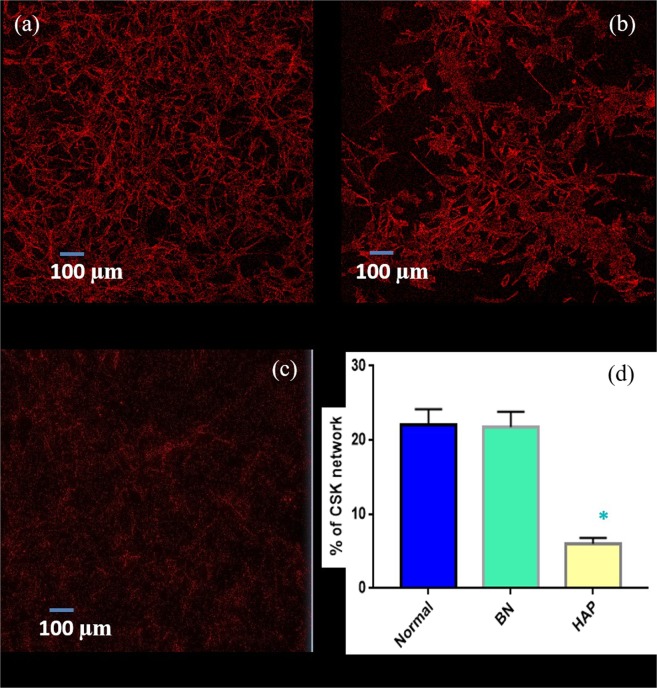
Confocal image of osteoblast CSK network for normal (**a**), BN NP (**b**) and HAP (**c**) treated cells. Cells were stained with Phalloidin to illuminate the F-actin network. (**d**) Quantative comparison of CSK network.

To further explore the potential mechanism for the reduction in adhesive property, focus was placed on cell cytoskeleton (CSK) network, which plays a vital role in cell adhesion, migration, embryogenesis, cell motility and cell orientation. The cells were stained with Alexa Fluor 568 Phalloidin to illuminate the F-actin network as red under confocal microscopy (Fig. 4a–c) in order to observe changes in the CSK network. A significant reduction in the F-actin network was observed in the HAP treated cells (22% for normal, 20% for BN treated cell and 6% for HAP treated cells). Furthermore, the F-actin network can be observed to be well connected in the normal cells; however, the filaments appear to be sparsely connected in HAP treated cells. This is a plausible reason for the drastic reduction in adhesive property of HAP treated cells at a concentration of 100 µg/ml.

### Effect of nanoparticle uptake on cell biochemical properties

**Figure 5:**
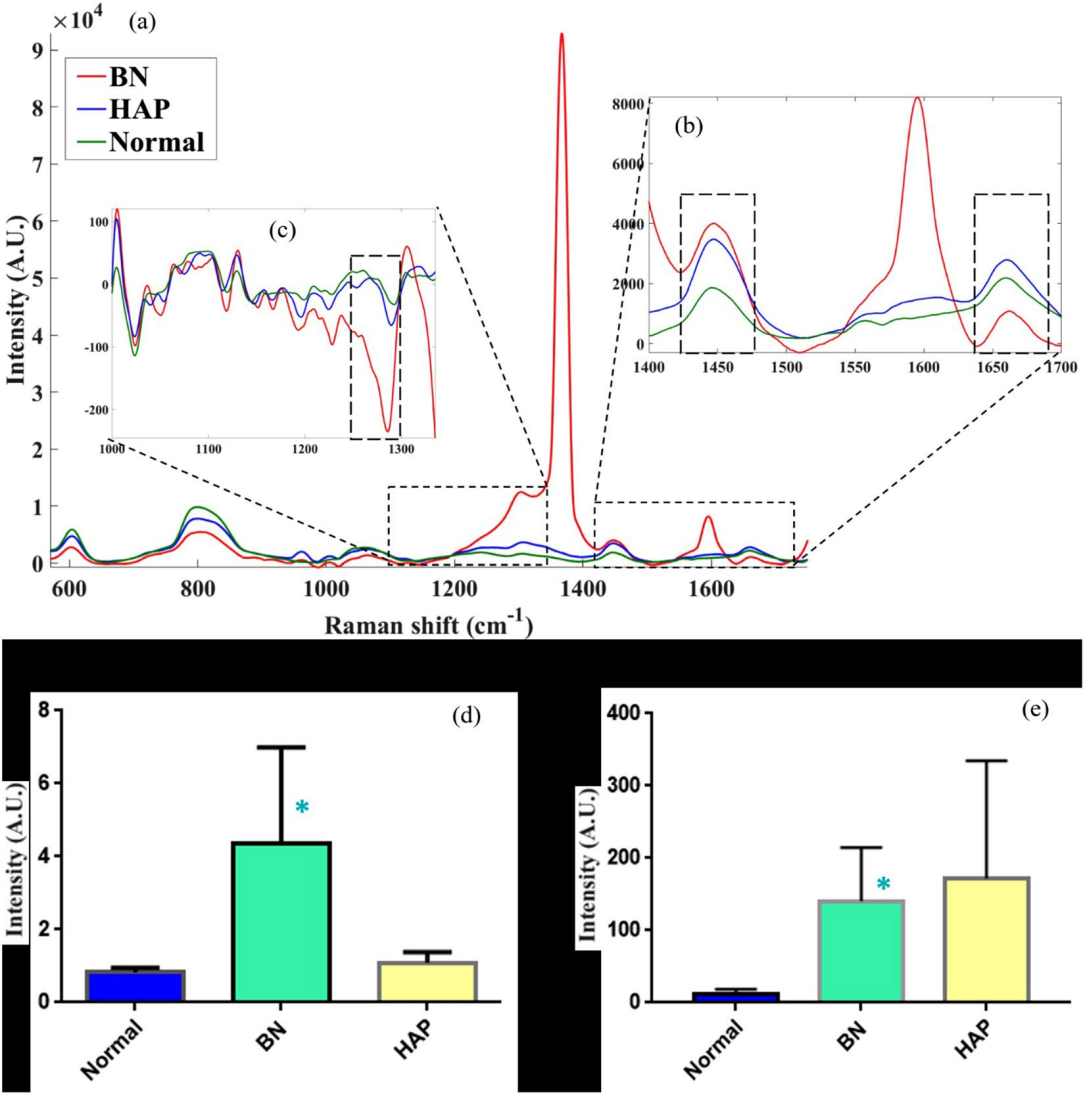
Raman spectra of human osteoblasts after culture in BN and HAP nanoparticles. (**a**) Full Raman spectra, with inset plot showing region (1400–1700 cm^−1^) with consistent changes between normal and treated cells. (**b**) Ratio of Raman peak intensity at 1400 cm^−1^ and 1660 cm^−1^. (**c**) Amide III peaks extracted via (1st) derivative transformation at 1280 cm^−1^. (**d**) Ratio of peaks at 1440 and 1660 cm^−1^. (**e**) lipid-associated peak at 968 cm^−1^.

The inconsistency in cell membrane post nanoparticle uptake has been clearly observed in Fig. 2. To further explore the effect, focus was placed on membrane biochemical composition. The feasibility of Raman spectroscopy for characterising disruptions in cell membrane has been effectively demonstrated^46^. As Raman spectroscopy responds to a range of active biological compounds, rather than single elements, it provides a suitable approach for investigating potential bulk biochemical changes in cells. Raman spectra were acquired from 600–1700 cm^−1^ to capture changes in cell membrane protein, lipids and nucleic acids. Considerable differences can be observed in the average Raman spectra of normal, BN and HAP treated cells (Fig. 5a). Changes in the spectral characteristics and peaks are arguably associated with corresponding biochemical changes in the cell membrane. Thus, the Raman spectra reveal the effect of (BN and HAP) nanoparticle uptake and interaction on the cell membrane.

The peak at 1440 cm^−1^ (Fig. 5b) is representative of CH_2_ bending and deformation vibrations, which is associated with functional groups in amino acid side chains of proteins (such as collagen) and lipids. The peak at 1660 cm^−1^ represents amide I vibration mode of structural proteins (C = C stretching vibration) associated with collagen-like proteins^31^ and unsaturated fatty acids. Thus, the ratio of these peaks (at 1440 and 1660 cm^−1^, Fig. 5d), possibly indicative of overall integrity of the cell membrane, revealed significant (p = 0.0177) effect of nanoparticle uptake on the treated cells, although no significant difference was observed based on the individual peaks. The ratio varied from 0.7 to 1.03 for normal cells, 0.76 to 1.4 for HAP treated cells, and for 1.6 to 7.6 BN treated cells.

Amide III peak (around 1280 cm^−1^), which is strongly indicative of the collagen macromolecules, possibly within the CSK, was extracted via (1^st^) derivative transformation to minimize the strong BN absorption (at 1380 cm^−1^). Significant difference (p = 0.013) was observed between normal and treated cells, with BN treated cells exhibiting significantly larger difference compared to normal cells (Fig. 5c), suggesting significant alteration in the cell membrane structural integrity. This is likely the reason for the increased stiffness observed in BN treated cells.

To further assess the effect of nanoparticle interaction with cells, alteration of the cell membrane lipids was investigated by observing changes in the lipid-associated peak at 968 cm^−1^ (Fig. 5d). Statistically significant difference (p = 0.005) was observed in the cells after BN treatment relative to normal cells; however, less significant difference (p = 0.059) was observed in HAP treated cells. The large variation in the lipid peak of HAP treated cells is consistent with HAP affinity for lipids, which can be used to separate different lipids^47,48^. The influence of nanoparticle uptake on cell nucleic acid can be observed in the peak at 800 cm^−1^. Consistent and statistically significant decrease (p < 0.0001) in this peak from normal to HAP and BN, respectively, can be observed (Fig. 4a). As with collagen macromolecule, the changes observed suggest substantial modification of the fundamental structure of the cells after nanoparticle uptake.

## Conclusion

In this study, we investigated the effect of nanoparticle uptake on the biophysical properties and response of cells. AFM images of osteoblasts indicated change in cell membrane profile after nanoparticle uptake. It was further observed that, local mechanical properties and overall cell adhesive properties of cells were also affected. Uptake of BN and HAP nanoparticles resulted in increased cell stiffness with different concentration and cellular region, while ingestion of HAP nanoparticles decreased cell adhesion at 100 µg/ml. In addition, Raman spectroscopy revealed significant changes in cell membrane biochemical properties and response, including lipids, protein and nucleic acid related changes after nanoparticle uptake. The outcome of this study opens up new insights into nanoparticles interactions with biological entities, resulting in better understanding of nanoparticle toxicity and applications.

## Methods and Materials

### Sample Collection and Experimental Methods

The experiments were conducted according to the Good Clinical Practice guidelines (Integrated Addendum To Ich E6, Nov 2016). Human osteoblasts were isolated from bone specimens collected from trauma patients who were undergoing above the knee amputations (age: 60.1 ± 6 years). The Human Research Ethics Committee of Queensland University of Technology (QUT) and the Prince Charles Hospital approved this study and the participants’ informed consent was obtained according to the Declaration of Helsinki (approval number: 1400001024). All experimental protocols were formally evaluated and approved by faculty and QUT health, safety and environment committee.

### Preparation of the Nanomaterials

The BN NP’s were collected from Momentive Performance Materials Inc. (USA) whereas the HAP nanoparticles were synthesised locally (QUT)^49^. The solution was prepared by diluting powders into UHQ waters. Thereafter, the solution was ultrasonicated using a needle based ultrasonicator (Misonix Sonicator, 3000) for 4 h at ambient temperature. Finally the solution was sterilized by autoclaving at 121 °C for 20 mins before conducting any cell culture work.

### Cell Culture

Osteoblast cells were cultured in six well plates using Dulbecco’s Modified Eagle’s Medium (low glucose) (GIBCO, Invitrogen Corporation, Melbourne, Australia) supplemented with 10% fetal bovine serum (FBS) (HyClone, Logon, UT) and 1% penicillin and streptomycin (P/S) (GIBCO, Invitrogen Corporation, Melbourne, Australia). They were stored in a secure environment depicting the human body condition (37 °C in a humidified atmosphere with 6% CO2) allowing them to grow. Once the cells were confluents, they were detached using 0.5% Trypsin (Sigma-Aldrich) and distributed into necessary plates or dishes depending on the study performed (AFM, TEM, Confocal etc.).

### Transmission Electron Microscopy

Cells were cultured with BN NP and HAP for different time periods (8, 12, and 24 hrs) according to the experimental conditions. They were then treated in a step-by-step manner (Buffer wash, post fixation, dehydration, and resin implantation). The cultured cells were fixed using 3% Glutaraldehyde (10 min) and rinsed twice using 0.1 M Cacodylate buffer (20 min). Then they were treated with 1% Osmium Tetroxide for an hour. They were rinsed again with UHQ water thrice (10 min each) before being treated with 1% Uranyl Acetate for an hour. Cells were then dehydrated by multiple exchange of ethanol, using progressively higher ethanol concentration starting from 50% to 100%. This was followed by resin (LX112) implantation in the culture dish. The concentration of the resin was varied from a low to high resin ethanol ratio (1:2, 1:1, 2:1). Finally the cells were treated with 100% percent resin (1 h) before being placed in the oven to be moulded at 70 °C (24 hrs).

The processed samples were then sectioned in 80 nm thickness using the Leica UC7 Ultramicrotome. The sectioned samples were placed in a 400 mesh carbon grids and taken to JEOL TEM 1400 for imaging. Samples were then carefully placed in the TEM holder and secured; making sure they would not be displaced inside the microscope.

### Scanning Electron Microscopy

Tescan Mira 3 was used for taking SEM images of BN and HAP nanoparticle as well as of cells after nanoparticle uptake. Negative voltage biasing of the sample stage and an In-beam detector that works as secondary electron detector in the beam deceleration mode (BDM) was used for imaging BN. −5kV stage bias and 5 kV landing energy with a view field of 3.95 µm was used. HAP nanoparticles and processed cultured cells were gold coated and imaged under secondary electron detector with 10 kV for 15 kV voltage.

### Confocal Microscopy

After the cells were cultured with BN NP and HAP for a certain period of time, the cells were gently washed with PBS (Sigma-Aldrich). The cells were then fixed with 4% paraformaldehyde (Sigma-Aldrich) for 20 min. The samples were repeatedly washed in PBS for several minutes in order to remove any unwanted reagents. Cells were then permeabilized with 0.1% Triton X100 (Sigma-Aldrich). After a few more washes with PBS, the samples were then incubated with 1:100 of DAPI and Alexa Fluor 568 Phalloidin (GIBCO, Invitrogen Corporation, Melbourne, Australia) for 10–15 min in order to stain the nuclei and actin filament networks respectively. The samples were then washed one more time and were taken to a confocal microscope (Nikon A1R confocal, Japan) for imaging. The 20x or 40x oil immersion objective lens were used depending on experimental necessity.

### Mechanical Property Quantification

**Figure 6:**
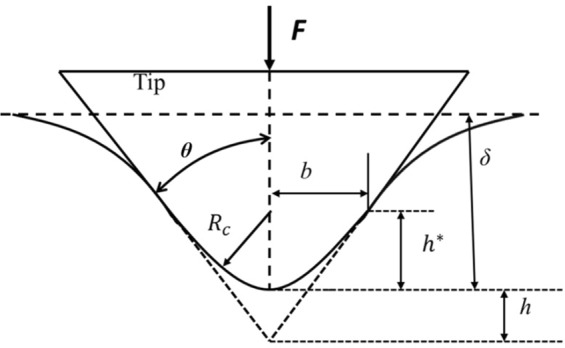
A schematic of the blunted pyramidal AFM tip. The semi-included angles is expressed as *θ*, tip defect as *h*, spherical cap radius as *R*_*c*_, the radial distance while transitioning from spherical caps to pyramidal face, the applied force as *F* and the indentation as *δ*.

The force indentation (*F-δ*) relationship for a pyramidal cantilever tip was developed using the Betti’s reciprocal theorem^50^. A schematic diagram of the tip indicating different key parameters are given in Fig. 6. The total applied force by the cantilever tip on a projected area A was calculated from the pressure distribution of the flat indenter ${P}^{\ast }(r,\phi )$ and can be expressed as^32^,

1 $$F=\int \,\,{\int }_{A}\,{P}^{\ast }(r,\phi )\frac{\int \,(r,\phi )}{{\delta }^{\ast }}rdrd\phi $$

Where,

δ^*^ = indentation depth and

$\int \,(r,\phi )$ = interpenetration function of the tip

${P}^{\ast }(r,\phi )$ and *F* was approximated using the best elliptical approximation of the contact area. For a pyramidal tip, the best elliptical approximation would be a circle with a radius *a* and therefore, the pressure distribution can be expressed as^32^,

2 $${p}^{\ast }(r,\phi )=\frac{E}{\pi (1-{v}^{2})}\frac{{\delta }^{\ast }}{{({a}^{2}-{r}^{2})}^{1/2}}$$

Therefore, Equ. 1 can be expressed as,

3 $$F=\frac{E}{\pi (1-{v}^{2})}\int {\int }_{A}\frac{\int (r,\phi )}{{({a}^{2}-{r}^{2})}^{1/2}}rdrd\phi $$

Where,

E = Young’s modulus

*v* = Poisson’s ratio

The pyramidal tip was slowly remodelled from a spherical to a pyramidal tip in accordance with the following function,

$$\int (r)=\delta -\frac{{r}^{2}}{2{R}_{c}}$$

4 $$f(r,\phi )=\delta -\frac{r-b}{\tan \,\theta }\,\cos (\phi -\frac{2k\pi }{n})-h$$

$$a\ge b,\frac{(2k-1)\pi }{n} < \phi  < \frac{(2k+1)\pi }{n},k=0,1,\mathrm{...........},n-1$$

Where,

*b* = radial distance in relation to transmission from spherical to pyramidal face,

5 $${h}^{\ast }={b}^{2}/2{R}_{c}$$

For *a* < *b*, *F*(*δ*) can be expressed as the spherical Hertz model

6 $$F=\frac{4E}{3(1-{v}^{2})}{R}_{c}^{1/2}{\delta }^{3/2}$$

For *a* > *b*, *F*(*δ*) can be expressed as the spherical Hertz model

7 $$F=\frac{1}{{2}^{1/2}}\frac{E\,\tan \,\theta }{(1-{v}^{2})}{\delta }^{2}$$

Here the effective radius contact a = *δ*tan*θ*/2^1/2^

### Adhesion Property Quantification

**Figure 7:**
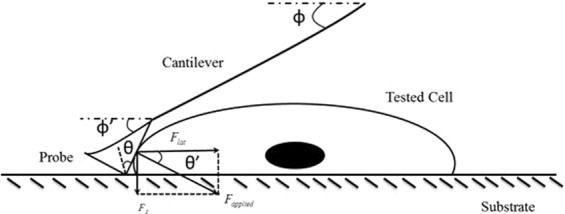
A schematic diagram of interaction between AFM cantilever tip and living cells while detaching cells from substrate.

The detachment process of cell using the AFM cantilever is illustrated in Fig. 7. To quantify the shear force required to detach individual cells from the substrate, Deupree *et al*. proposed a novel approach based on the total compression of the cantilever during the manual detachment event^43^. They expressed the lateral force F_lat_ as,

8 $${F}_{lat}=kS{V}_{total}\,\sin (\varphi +\theta )\cos \,\theta $$

Where,

*F*_*lat*_ = lateral detachment force

*k* = spring constant

*S* = sensitivity

*V*_*total*_ = *V*_*total*_ is the total vertical deflection of the reflected laser beam on the photodiode detector and

*ϕ* and *θ* = parameter related to cantilever probe geometry and cantilever orientation

However, Zhang *et al*. observed that due to the counterforce from the tested samples (cells) the cantilever will slightly bend when operated in contact mode and hence the angles *φ* and *θ* will change accordingly^44^. Therefore, Eq. 7.6 can be rewriter as,

9 $${F}_{lat}=kS{V}_{total}\,\sin (\varphi +\theta )\cos \theta ^{\prime} $$

The angle *ϕ*’ and *θ*’ can be expressed as^44^,

10 $$\varphi ^{\prime} =2\arctan \,[\frac{L-\sqrt{{({V}_{total}S)}^{2}+{(L\cos \phi )}^{2}}}{{V}_{total}S+L\,\sin \,\varphi }]$$

11 $$\theta ^{\prime} =\phi +\theta -2\arctan \,[\frac{L-\sqrt{{({V}_{total}S)}^{2}+{(L\cos \varphi )}^{2}}}{{V}_{total}S+L\,\sin \,\varphi }]$$

Here, *L* = Applied cantilever length.

Finally, from Eq. 7.7 the lateral force *F*_*lat*_ can be established as^44^ which was used for the lateral force quantification,

12 $${F}_{lat}=kS{V}_{total}\,\sin (\varphi +\theta )\times \,\cos \,\{\varphi +\theta -2\,\arctan \,[\frac{L-\sqrt{{({V}_{total}S)}^{2}+{(L\cos \varphi )}^{2}}}{{V}_{total}S+L\,\sin \,\varphi }]\}$$

Cells were cultured in a petri dish with both BN NP and HAP with a concentration of 50 and 100 µg/ml. For each experiment, all the tested cells were from the same batch and were subjected to the exact same culture environment to avoid external influence on the measured forces. Prior to the experiment, the spring constant was confirmed (7.6012 N/m) by conducting a thermal tuning. The image size was set to 100 × 100 µm with 2 s per line. Once the scanline is set, the cantilever was moved to the centre of the cell therefore displacing it from the substrate. From the deflection of the cantilever, maximum lateral force was quantified.

### Raman Spectroscopy

Raman measurements were collected using an in Via-Raman microscope (Renishaw, UK), equipped with a 1200 l/mm grating. A 785 nm laser was used for excitation, providing 1 mW laser power at the sample. The laser was brought to a line focus of approximately 0.8 × 15 μm using an x50 Leica N Plan objective (NA 0.75). Raman scattering was detected with a Renishaw CCD camera with 40 s exposure time and 16 accumulations generated by WiRE2 spectral acquisition software. All measurements were carried out under ambient conditions and instrumentation was calibrated to the 520.5 cm^−1^ line of Si prior to the actual experiments. A total of 10 cells per sample were measured.

## Supplementary information

Supplementary Materials
