# Supplementary material for: Impact of Nanoparticle Uptake on the Biophysical Properties of Cell for Biomedical Engineering Applications
Source: Sci Rep. 2019 Apr 10;9:5859. doi: 10.1038/s41598-019-42225-7 (PMC6458124; doi:10.1038/s41598-019-42225-7)
Supplement: Supplementary file 1 — Supplementary Materials [file 41598_2019_42225_MOESM1_ESM.pdf]

Supplementary Materials

Impact of Nanoparticle Uptake on the Biophysical Properties of Cell for Biomedical Engineering Applications

Md. Alim Iftekhar Rasel[1]

Sanjleena Singh[1]

Trung Dung Nguyen[2]

Isaac O Afara[3]

Yuantong Gu[1, *]

[1] School of Chemistry, Physics and Mechanical Engineering, Queensland University of Technology (QUT), Brisbane, Australia

[2]Department of Aerospace and Mechanical Engineering, College of Engineering, University of Notre Dame, Notre Dame, Indiana 46556, USA

[3] Department of Applied Physics, University of Eastern Finland, Kuopio, Finland.

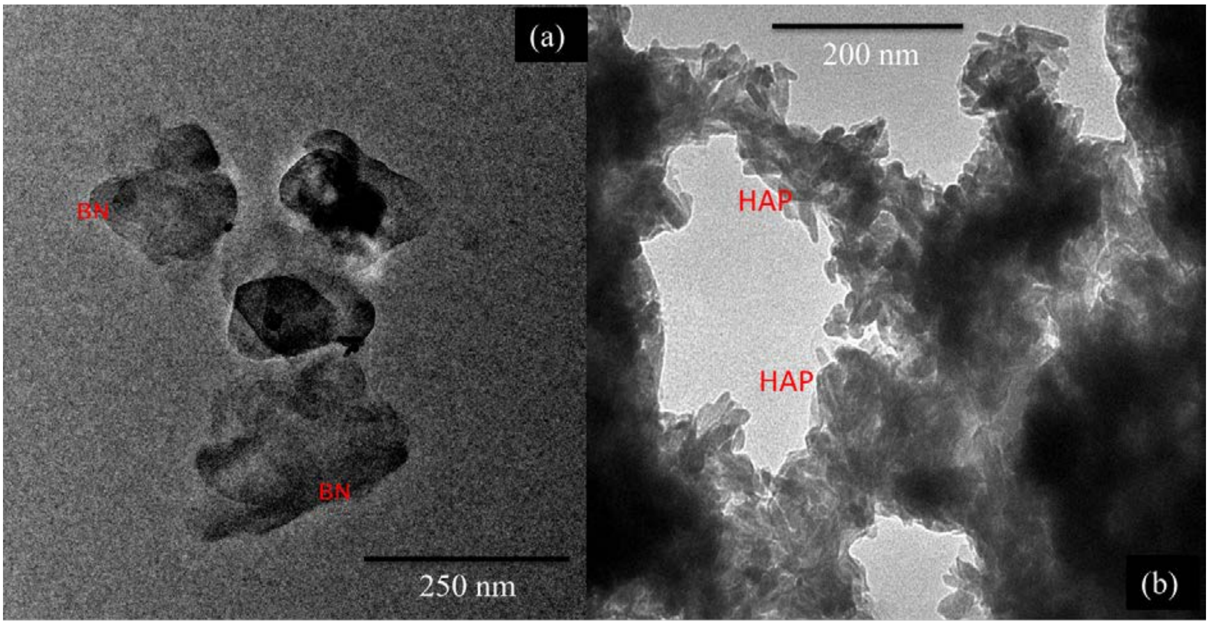

Figure 1 TEM images of BN (a) and HAP (b). The size of BN range from 150-250 nm while for HAP it was 40-50nm. HAP’s are observed to be heavily aggregate. In comparison little aggregation is observed for BN.

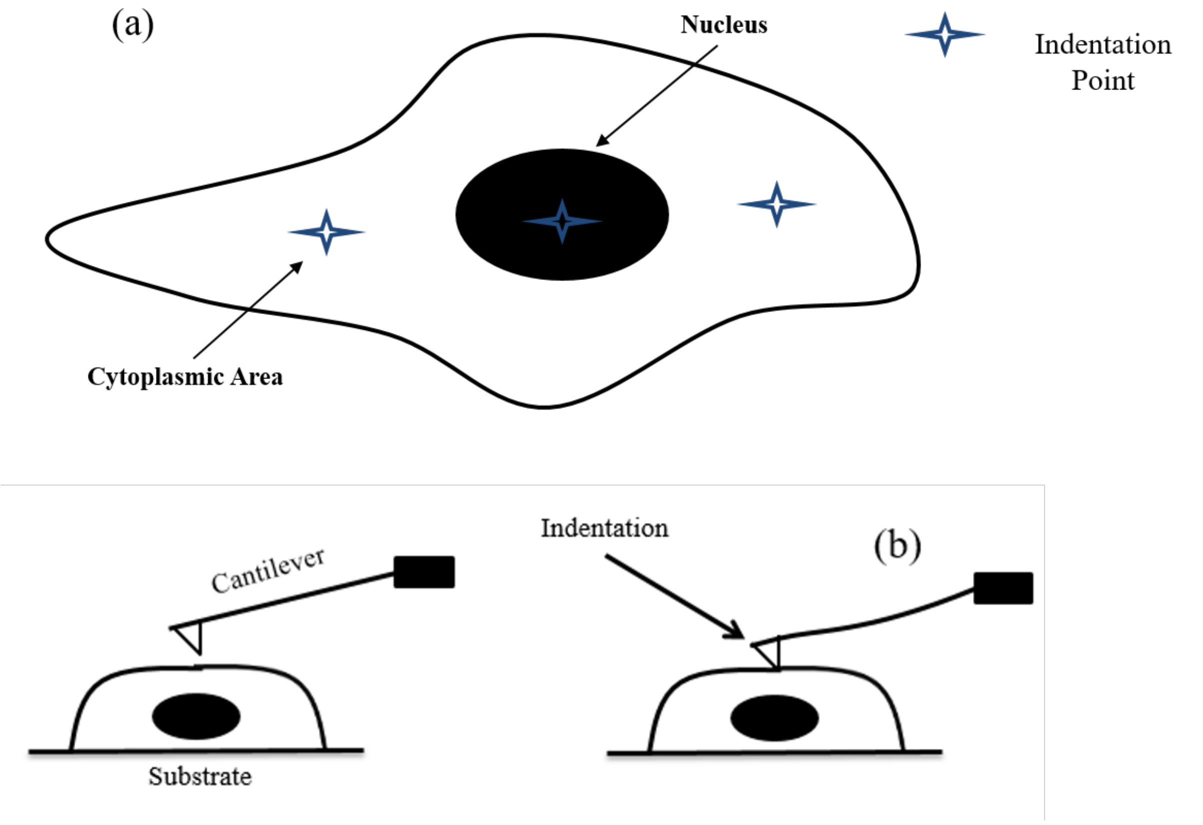

Figure 2 : A schematic outlining the indentation sites. Indentation is conducted both in the cytoplasmic region as well as on the nucleus.
